# COVID-19: A Brief Overview of the Discovery Clinical Trial

**DOI:** 10.3390/ph13040065

**Published:** 2020-04-10

**Authors:** Jean Jacques Vanden Eynde

**Affiliations:** Formerly head of the Department of Organic Chemistry (FS), University of Mons-UMONS, 7000 Mons, Belgium; jean-jacques.vandeneynde@ex.umons.ac.be

**Keywords:** chloroquine, COVID-19, hydroxychloroquine, lopinavir/ritonavir, remdesivir, repurposing, SARS-CoV-2

## Abstract

The outbreak of COVID-19 is leading to a tremendous search for curative treatments. The urgency of the situation favors a repurposing of active drugs but not only antivirals. This short communication focuses on four treatments recommended by WHO and included in the first clinical trial of the European Discovery project.

## 1. Introduction

COVID-19 (coronavirus disease 2019) is a worldwide outbreak of pneumonia and acute respiratory distress syndrome. It is unambiguously linked to the human–human transmission of a novel coronavirus. Mutagenomic analyses [1] demonstrated that 79.6% of the genome sequences were identical to those found in the coronavirus responsible for the severe acute respiratory syndrome (SARS), which spread in 2002. Hence the classification as 2019-nCoV (n for novel) and the name SARS-CoV-2 [2].

Combatting SARS-CoV-2 has emerged as a tremendous challenge, gathering efforts from academia, pharmaceutical companies, hospitals, international organizations, as well as governments and philanthropic associations (e.g., The Council on Foundations [3]). In this *Opinion*, we give a brief overview of some current and future clinical trials, as they can be found in the U.S. National Library of Medicine of the National Institutes of Health (Bethesda, MD, USA) [4].

## 2. Current Clinical Trials: An Overview

As of March 28, 202 clinical trials could be retrieved with the search term “COVID-19” in the database [4] of the U.S. National Library of Medicine. The number jumped to 388 as of April 8. Among these trials are:

- Seven studies involving human plasma;
- 11 studies involving traditional Chinese medicine (TMC);
- 14 studies involving stem cells, mostly mesenchymal stem cells;
- 16 studies involving dietary complements, including vitamin C, and honey;
- 27 studies dedicated to vaccines;
- 52 studies involving proteins, including commercially available monoclonal antibodies;
- 70 studies involving antiviral drugs;
- More than 100 studies involving other small molecules.

A quick reading of these projects [4] allows one to sort out the monoclonal antibodies, antiviral drugs, and other small molecules that are most cited in the trials. The list can be found in Table 1.

## 3. The SOLIDARITY Response Fund and the Discovery Project

On March 13, the United Nations Foundation, the Swiss Philanthropy Foundation, and the World Health Organization (WHO) have created the SOLIDARITY Response Fund in order to raise money to support studies on COVID-19 [5].

On March 18, WHO indicated that the first trial supported by the Fund would be an adaptative study performed in ten countries, namely Argentina, Bahrain, Canada, France, Iran, Norway, South Africa, Spain, Switzerland, Thailand. India joined the trial on March 27.

On March 22, the French Institut National de la Santé Et de la Recherche Médicale (INSERM) announced a European counterpart, named Discovery, and aimed at a study of four treatments on 3100 patients in seven countries, namely France, Spain, the United Kingdom, Germany, Luxembourg, the Netherlands, and Belgium [6]. The four treatments to be analyzed are:

- Remdesivir;
- The combination lopinavir/ritonavir;
- The combination lopinavir/ritonavir with the addition of interferon β-1a;
- Hydroxychloroquine, eventually associated with an antibiotic (azithromycin) in add-on studies.

The study is entitled “Trial of Treatments for COVID-19 in Hospitalized Adults (DisCoVeRy)” (NCT04315948) [4]. It started in three French hospitals: Centre Hospitalier Régional Universitaire de Lille (Lille, France), Centre Hospitalier Universitaire de Nantes (Nantes, France), and Assistance Publique Hôpitaux de Paris - Bichat Claude Bernard (Paris, France).

**Figure 1:**
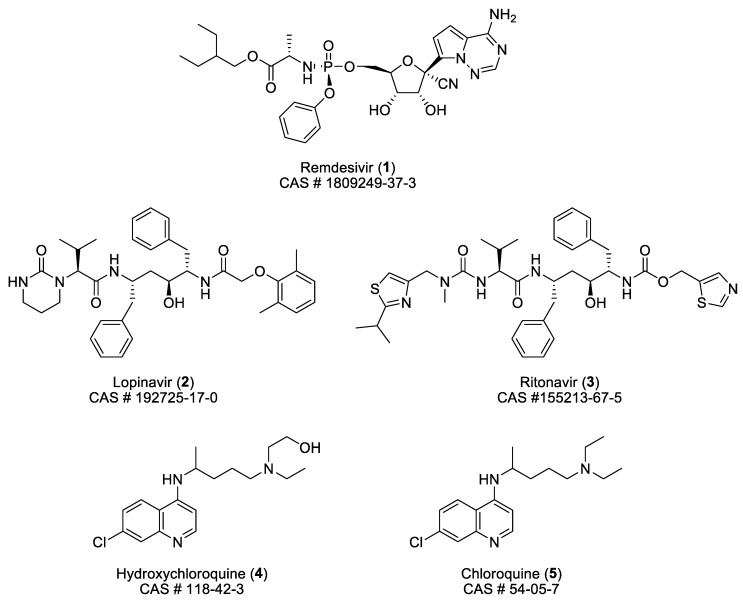
Structure and some characteristics of remdesivir (**1**), lopinavir (**2**), ritonavir (**3**), hydroxychloroquine (**4**), and chloroquine (**5**).

The structure and some characteristics of the four small molecules involved in that clinical trial are collected in Figure 1. The formula of chloroquine is added for comparison. 

## 4. Some Comments on the Treatments Suggested in the Discovery Project

It has been announced that the Discovery project is an adaptive study. That means that some treatments can be abandoned during the study if results are not encouraging, whereas new treatments could be added. Some noticeable characteristics of each of the four actual treatments are given in Table 2.

### 4.1. Remdesivir (**1**)

Remdesivir is an investigational drug developed by Gilead Sciences. Before the outbreak of SARS-CoV-2, it has been the subject of only one clinical trial, following the U.S. National Library of Medicine of the National Institutes of Health [4]. That study (NCT03719586) was entitled “Investigational Therapeutics for the Treatment of People with Ebola Virus Disease” and started on November 21, 2018. Three monoclonal antibodies (ZMapp for the control group, REGN-EB3, Mab114) and remdesivir were separately administered to a total of 673 participants. An initial dose of 200 mg of remdesivir was administered on day 1, followed by a daily maintenance dose of 100 mg for 9–13 days. Results were disappointing. After 28 days, the highest number of deaths (53.1%) was observed in the group treated with remdesivir, a bit more than in the control group (49.7%). More encouraging results were obtained in the two other groups (REGN-EB3: 33.5 %; Mab114: 35.1%) [7].

In contrast, in the cases of SARS-CoV and MERS-CoV, remdesivir exhibited an excellent activity, in vitro, in the submicromolar range with EC_50_ values of 0.07 μM for both coronaviruses [8]. Results were confirmed in vivo in the case of an infection by MERS-CoV in mice [9] and rhesus macaque models [10]. However, the conclusion of Sheahan [9] regarding SARS-CoV in a mouse model warned that “These data suggest that reductions in viral load after peak lung titers were achieved were insufficient to improve outcomes after the immunopathological phase of disease had been initiated. Thus, in the mouse, if given prior to the peak of SARS-CoV replication and peak damage to the airway epithelium, GS-5734 (remdesivir) can improve pulmonary function, reduce viral loads and diminish disease.”

An EC_50_ value of 1.76 μM for remdesivir against SARS-CoV-2 in Vero E6 cells has been measured by Wang et al. [11]. There is a study involving only one human: the first patient suffering from COVID-19 in the United States [12]. He was hospitalized on January 19 in the State of Washington. For the first 6 days of hospitalization (days 5–10 of the illness), he received 650 mg of acetaminophen every 4 h, 600 mg of ibuprofen every 6 h, 600 mg of guaifenesin and 6 liters of normal saline. On day 6 of hospitalization, he was treated with vancomycin (1750 mg then 1 g every 8 h) and cefepime (every 8 h). The next day, remdesivir was administered for compassionate use, whereas the antibiotics were discontinued. The authors concluded their article by writing [12]: “On hospital day 8 (illness day 12), the patient’s clinical condition improved. Supplemental oxygen was discontinued, and his oxygen saturation values improved to 94% to 96% while he was breathing ambient air. The previous bilateral lower-lobe rales were no longer present. His appetite improved, and he was asymptomatic aside from intermittent dry cough and rhinorrhea. As of January 30, 2020, the patient remains hospitalized. He is afebrile, and all symptoms have resolved with the exception of his cough, which is decreasing in severity. … Although a decision to administer remdesivir for compassionate use was based on the case patient’s worsening clinical status, randomized controlled trials are needed to determine the safety and efficacy of remdesivir and any other investigational agents for treatment of patients with 2019-nCoV infection.”.

It is actually too early to calculate the price of an eventual treatment of COVID-19 with remdesivir, but some thoughts can be explored. The dosage that will be evaluated in the Discovery project (NCT04315948 [4,13]) is 200 mg IV initially, then 100 mg OD for 2–10 days. The product, as a research derivative, is actually sold at 854 USD for 10 mg by MedChemExpress (Monmouth Junction, NJ, USA; cat # HY-104077) [14] and 330 USD for 1 mg by BioVision Inc (Milpitas, CA, USA; cat # B2997-1000) [15], it is not pessimistic to suggest a retail price higher than 5000 USD.

### 4.2. The Combination Lopinavir/Ritonavir (**2**/**3**)

The commercial combination (Kelatra®) comprises lopinavir and ritonavir in a weight ratio 4:1; it is available for the treatment of HIV infection. It has been the subject of more than 400 registered clinical trials [4].

In vitro, the combination is more than 100-fold less effective than remdesivir against strains of SARS-CoV (EC_50_ = 17.1 μM [16]) and MERS-CoV (EC_50_ = 8.0 μM [16]). Other data indicated some success regarding the use of the combination in marmosets (MERS) [17] and in infected human patients (SARS as well as MERS) [18]. However, some authors remain skeptical and are not confident in the success of Kelatra® for treating COVID-19 [19,20,21,22]. 

In the Discovery project (NCT04315948 [4,13]), the suggested dose of the combination is 500 mg (400 mg lopinavir + 100 mg ritonavir) every 12 h for 14 days. On the basis of the lowest retail price found on the website PharmacyChercker.com [23], the cost of the treatment, if the drug combination proves to be efficacious in humans, should be estimated at 61 USD for the generic and 215 USD for the original brand.

### 4.3. The Combination Lopinavir/Ritonavir with the Addition of Interferon β-1a (**2**/**3**/INF β-1a) 

Interferon β-1a is an efficient inhibitor of the multiplication of SARS-CoV [24,25] and MERS-CoV [26] in cell cultures. In a MERS-CoV mouse model, the combination lopinavir/ritonavir/interferon β-1a, used as prophylactic and curative treatments, revealed no significant decrease in the viral load [9]. In a marmoset model [17], the combination lopinavir/ritonavir/interferon β-1b (bacterial origin, whereas interferon β-1a is produced by Chinese hamster ovary cells [27]) opened some hope for curing MERS-CoV-infected animals. The results of an ongoing clinical trial (NCT 02845843 [4]), entitled “MERS-CoV infection treated with a combination of lopinavir/ritonavir and interferon Beta-1b”, are eagerly awaited, but will not be disclosed before 2021. The study suggested a dose of lopinavir/ritonavir (400/100 mg) twice daily for 14 days, and interferon β-1b 0.25 mg subcutaneous every alternate day for 14 days [4].

Interferon β-1a is clinically used for the treatment of relapsing multiple sclerosis. It was approved by the FDA on 05/17/1996 under the brand name Avonex® and is sold by Biogen under the Orphan Drug designation. It is also known as Rebif® from Serono Inc (FDA-approved on 03/07/2002). In the Discovery project, interferon β-1a will be added to the treatment lopinavir/ritonavir (see Section 4.2) and administered subcutaneously at the dose of 44 µg for a total of three doses in 6 days. That will represent an additional cost of almost 2000 USD for Rebif®, following [23].

### 4.4. Hydroxychloroquine (**4**) and Chloroquine (**5**)

There is a huge confusion in the public, as well as among politicians and even some scientists, about those two molecules. That confusion ultimately led to people accidently overdosing [28].

Both molecules can be classified as 4-aminoquinolines, and both have anti-plasmodial activities, but they remain two different molecules with some different metabolites. Chloroquine has been widely used since the 1950s for prophylactic and curative treatments of malaria. Its extensive use has led to the emergence of chloroquine-resistant strains of *Plasmodium falciparum*, the parasite responsible for the severest form of malaria. Such strains are also resistant to the structurally related hydroxychloroquine. Regardless, the latter is sometimes preferred because of its lower (retinal) toxicity [29,30]. Chloroquine (Aralen®) is indicated for the treatment of malaria and extraintestinal amebiasis. Hydroxychloroquine (Plaquenil®) is also prescribed for the treatment of malaria. It is also used successfully to treat lupus erythematosus and rheumatoid arthritis [27]. In addition, it should be noted that chloroquine, essentially, and hydroxychloroquine, to a lesser extent, have been clinically tested for their anti-HIV activity [4,31].

As recently as 2006, the anti-coronavirus properties of hydroxychloroquine and chloroquine were reported by Biot et al. [32]. Those authors observed that, in vitro, both molecules were active against SARS-CoV with EC_50_ values of 34 and 6.5 μM, for hydroxyquinoline (**4**) and chloroquine (**5**), respectively. The difference is less marked in the study of Dyall et al. [33] who reported EC_50_ values of 7.97 μM (**4**) and 6.54 μM (**5**) against SARS-CoV and 8.28 μM (**4**) and 6.28 μM (**5**) against MERS-CoV. The trend is reversed when dealing with SARS-CoV-2, as values related by Yao et al. [34] were 0.72 μM (**4**) and 5.47 μM (**5**). A comparable efficacy of 6.9 μM was measured by Wang et al. [11] for chloroquine (**5**).

On February 19, Gao et al. [35] recorded, in China, 15 clinical trials involving chloroquine. On March 28, the FDA issued an Emergency Use Authorization for use of hydroxychloroquine and chloroquine for patients who do not have access to the drugs via clinical trials [36]. As underlined by a reviewer of this manuscript, this essentially means that lots of anecdotal testimonies as to the effects of these two drugs will be released until properly controlled clinical trials are done. As of 8 April, 58 clinical trials concern hydroxychloroquine, compared to 19 on March 28 [4]. In France, controversial results have been published, in March, on a treatment associating the same dosing regimen of hydroxychloroquine and azithromycin. The treatment was administered to small cohorts (less than 25 people) of patients hospitalized for severe COVID-19 infection [37,38]. Due to such confusion, in France and Belgium (and probably in other countries), politicians, their advisors, and even some physicians called for caution. They suggested that preliminary reports describing successful outcomes in the use of hydroxychloroquine or chloroquine in hospitals must be carefully considered, either because those two molecules are plagued by side-effects or because of a lack of scientific rigor in the studies. Contrastingly, The Bill and Melinda Gates foundation announced that it had launched and financially supported two trials aiming at a study on the use of hydroxychloroquine as a prophylactic agent for COVID-19 [39]. 

In the Discovery project, the suggested dosage for hydroxychloroquine is 400 mg for the initial dose, followed by 400 mg 12 h later and then 200 mg BID for up to 4 days. Using the same metric [23] to evaluate the cost of the treatment, the price should not exceed 4.1 USD. Alternatively, a higher dosage is proposed for chloroquine: 600 mg for the initial dose, followed by 600 mg 12 h later and then 300 mg BID for up to 4 days. Considering the commercial availability of chloroquine as a diphosphate salt, the retail price would not exceed 6.6 USD [23].

## 5. Conclusions

The first cases of COVID-19 emerged in Wuhan, China, in late 2019, and to date there is no approved specific drug to cure patients infected by SARS-CoV-2. Politicians and physicians suggest that we should wait for clinical trials’ data before considering an eventual treatment. In Europe, the Discovery project (NCT04315948) has a study start date of March 22, 2020 and will enroll 3100 patients. The first results should be available within time frames of 15 and 29 days, but no publication date has been announced. The estimated study completion date has been set in March 2023.

As of April 8, there are 388 ongoing studies on COVID-19 that are recorded in the U.S. National Library of Medicine of the National Institutes of Health [4]: a breath of hope. 

## Figures and Tables

**Table 1:** Most cited monoclonal antibodies, antiviral drugs, and other small molecules in COVID-19 clinical trials.

**Monoclonal antibodies**				
**Name**	**Number of trials as of April 8** **(as of March 28)**	**FDA-Approved**	**Brand name**	**Manufacturer(s)**
Tocilizumab	15 (6)	01/08/2010	Actemra	Roche
Sarilumab	5 (4)	05/22/2017	Kevzara	Sanofi and Regeneron
Bevacizumab	2 (2)	02/26/2004	Avastin	Roche
**Antiviral drugs**				
**Name**	**Number of trials**	**FDA-Approved**	**Brand name**	**Manufacturer(s)**
Lopinavir + Ritonavir	22 (14)	09/15/2000	Kaletra, Aluvia	AbbVie
Umifenovir	10 (9)	investigational	Arbidol, Abidol	Available in China and Russia
Remdesivir	9 (9)	investigational		Gilead Sciences
Oseltamivir	6 (4)	10/27/1999	Tamiflu	Gilead Sciences, Roche
ASC09 or TMC-310911	5 (3)	investigational		Janssen
Favipiravir	4 (2)	Approved in Japan (2014)	Avigan	Toyama Chem
**Other small molecules**				
**Name**	**Number of trials**	**FDA-Approved**	**Brand name**	**Manufacturer(s)**
Hydroxychloroquine	58 (19)	04/18/1955	Plaquenil	Sanofi
Chloroquine	23 (12)	04/18/1955	Aralen	Sanofi
Methylprednisolone	6 (5)	10/24/1957	Depo-Medrol, Solu-Medrol	several
Losartan	5 (2)	04/14/1995	Act Losartan Cozaar	Actavis Pharma several
Colchicine	4 (2)	07/27/1961	Colchicine	several
Thalidomide	2 (2)	07/16/1998*	Thalidomid *	Celgene
Baricitinib	2 (2)	05/31/2018	Olumiant	Eli Lilly & Co

* The product has been reintroduced in the market after it was withdrawn in 1961 due to its teratogenic effects (scandal of the Softenon babies).

**Table 2:** Some noticeable characteristics of the four treatments considered in the Discovery project.

Characteristics	Remdesivir	Lopinavir (2) Ritonavir (3)	2 + 3 + IFNβ-1a	Hydroxy-Chloroquine	Chloroquine
EC _50_ (μM) SARS-CoV	0.07 [8]	17.1 [16]	-	34 [32] 7.97 [33]	6.5 [32] 6.54 [33]
EC_50_ (μM) MERS-CoV	0.07 [8]	8.0 [16]	-	8.28 [33]	6.28 [33]
EC_50_ (μM) SARS-CoV-2	1.76 [11]	-	-	0.72 [34]	5.47 [34] 6.9 [11]
Total number of CT ^a^ [4] ^b^	12	410		297	229
Number of CT ^a^ for COVID-19 [4] ^b^	9	22	3	58	23
Dosage for COVID-19 treatment following NCT04315948 [4] and [13]	200 mg IV then 100 mg OD for 2–10 days	400 mg (2) and 100 mg (3) every 12 h for 14 days	Same treatment as 2/3 + 3 doses of 44 µg IFNβ in 6 days	400 mg then 400 mg 12 h later, then 200 mg BID for up to 4 days	600 mg then 300 mg 12 h later, then 300 mg BID for up to 4 days ^c^
Estimated retail price for the treatment (USD)	> 5,000	61 (generic) 215 (brand)	> 2,000	4.1	6.6 ^d^

^a^ CT for clinical trials; ^b^ As of April 8; ^c^ The dosage for chloroquine diphosphate is 600 mg, then 300 mg 12 h later, then 300 mg BID for up to 4 days; ^d^ Cost considering chloroquine diphosphate.
